# Effectiveness of mechanical traction in supine versus prone lying position for lumbosacral radiculopathy

**DOI:** 10.12669/pjms.37.5.4200

**Published:** 2021

**Authors:** Rehan Ramzan Khan, Saima Riaz, Sajid Rashid, Muhammad Sulman

**Affiliations:** 1 Dr. Rehan Ramzan Khan, MSPT-OMPT. Assistant Professor, Multan College of Physiotherapy, Multan Medical and Dental College (MMDC), Multan, Pakistan; 2 Dr. Saima Riaz, MSPT-OMPT, PhD Assistant Professor, Riphah College of Rehabilitation & Allied Health Sciences, Riphah International University (RIU), Lahore, Pakistan; 3 Prof. Dr. Sajid Rashid, PhD. Multan College of Physiotherapy, Multan Medical and Dental College (MMDC), Multan, Pakistan; 4 Dr. Muhammad Sulman, MSPT-OMPT. Senior Lecturer, Department of Physiotherapy, University of Sialkot, Sialkot, Pakistan

**Keywords:** Radiculopathy, Mechanical Traction, Lumbar Spine

## Abstract

**Objectives:**

To determine the effectiveness of mechanical traction in supine versus prone lying position for lumbosacral radiculopathy.

**Methods:**

A quasi experimental trial was conducted from April to September 2020 among sixty patients of chronic lumbosacral radiculopathy at Ibn e Siena Hospital, Multan. Participants were divided into two groups. Group-A (Supine) participants received lumbar traction in supine lying along with conventional treatment. Group-B (Prone) underwent the same treatment except the lumbar traction being applied in prone lying position. Participants were evaluated twice: at pre- treatment (week 0) and at the post treatment (week 2). Oswestry Disability Index and Numeric Pain Rating Scale were used as outcome measure. Data was analyzed on SPSS 23.

**Results:**

The mean (±S.D) age of the patients was 39±5.7 vs. 40±5.3 years in supine vs. prone group respectively. Mean ODI score was 25.2±6.13 vs. 26.0±6.26 at the start of treatment in supine vs. prone position respectively while it was 19.45±7.12 vs. 11.05±4.40 at end of treatment in supine vs. prone position respectively. Mean NPRS score was 7.73±1.23 vs. 7.67±0.96 at start of treatment in supine vs. prone position respectively while it was 4.63±0.89 vs. 3.13±0.90 at the end of treatment in supine vs. prone position respectively.

**Conclusion:**

Lumbar traction in prone lying position is more effective than lumbar traction in supine lying position for the treatment of chronic lumbosacral radiculopathy.

## INTRODUCTION

Radiculopathy is a disorder arising from compression, impingement, inflammation or irritation of a spinal nerve root, which might be because of a disc herniation or any other local degenerative tissue encroaching the intervertebral foramen space.^1^ Lumbosacral radiculopathy is the clinical term used to depict an anticipated cluster of symptoms resulting from mechanical or inflammatory lesion affecting at least one of the lumbosacral nerve roots. Depending on severity, the presenting complaints can vary from radiating pain to paraesthesia, tingling sensations, muscular weakness and gait abnormalities. Contingent upon the nerve root(s) affected, clinical manifestations may correspond with the dermatomal/myotomal pattern.^2^

Lumbosacral radiculopathy causes pain in the lower back and hip which radiates down the back of the thigh into the leg. It is mostly caused by damage to one of the lower vertebra, ranging from L1 to S1.^2^ Lumbosacral radiculopathy occurs when spinal nerves roots are impinged by any of the conditions including central canal stenosis, disc protrusion, spondylolisthesis and other degenerative conditions.^3^

Radiculopathy of lumbosacral spine is a condition associated with important socioeconomic results. Out of 12.9% incidence of lower back pain among workers, 11% is due to lumbosacral radiculopathy. Frequency of radiculopathy in lumbosacral region approximates from 9.9- 25%.^4^

Physical therapy intervention includes pain relief modalities, muscle conditioning, stretching, lumbar traction and postural correction awareness leading to functional exercises.^5^ As radicular pain is due to encroachment of spinal canal causing compression on spinal nerve, lumbar traction alleviates this pain by vertebral separation causing decompression on pinched nerve.^6^

The application of continuous, intermittent, manual, mechanical, manual and sustained spinal traction have been employed to address pain complaints since the era of Hippocrates. It can be applied in different positions including supine and prone lying positions.^7^ Benefits of traction in lumbar region as explained by Cyriax include expansion of space between vertebrae and stretching of the posterior longitudinal ligament to generate pulling force to draw the herniated disc towards the central aspect of the joint. Various impacts associated with traction in lumbar region incorporate production of distracting force among facet joints and expansion of foraminal space.^8^ Traction has also been observed to decrease nucleus pulposus pressure^9^,^10^ and increase foraminal space.^10^

There are very few studies to compare the effectiveness of lumbar traction in supine and prone lying position for the treatment of lumbosacral radiculopathy. Therefore, this study was conducted to find out the best position between the two. The patients will benefit in better relief of symptoms if the lumbar traction is applied in position that is upheld by evidence for the management of lumbosacral radiculopathy. The results of this study will be useful for the Physical Therapists in planning and picking best treatment position for the utilization of lumbar traction in lumbosacral radiculopathy.

## METHODS

A quasi experimental trial was conducted from April to September 2020 to compare the effects of mechanical lumbar traction in supine and prone lying position for managing lumbosacral radiculopathy. A sample size of 60 patients was selected through online Epitool sample size calculator. Patients of both genders in age group of 20-50 suffering with chronic lumbosacral radiculopathy, sensory disturbances along L4-S1 dermatome, myotomal weakness along L4-S1 myotome and weak or absent patellar and ankle reflex were included in this study while patients with red flags, previous spinal surgery and spinal deformities were not included. Straight leg raise, reverse straight leg raise, slump test and crossed straight leg raise were the physical tests performed to confirm diagnosis. The participants were divided into two groups. Group A- Supine (n= 30) participants were provided with conventional physiotherapy treatment including moist heat, Transcutaneous Electric Nerve Stimulation (TENS) and lumbar traction in supine lying. The mode of lumbar traction was Intermittent with traction force 50% of body weight. The duration of lumbar traction for each session was 20 minutes with frequency of five sessions per week. The participants of group B- Prone (n= 30) were given the same treatment while applying lumbar traction in prone lying position. Assessment was done twice at pre-treatment (week 0) and post-treatment (week 3). ITO TM 400 traction system was employed to apply lumbar traction.

This research took place at Ibn-e-Siena Hospital and Research Institute, Multan with approval from ethical review board vide letter no. C-12-0320, Dated 12.03.2020. Convenience sampling technique was employed. Standardized outcome measures including Oswestry Disability Index and Numeric Pain Rating Scale were used for data collection before and after the treatment.

Data was analyzed by using SPSS version 23. Frequency percentage, mean and standard deviation were used for presentation of categorical and demographic features. The level of significance was accepted as P<.05. Numeric variables were defined as mean ± standard deviation. The normality of hypothesis was assessed using the Shapiro-Wilk test. Paired samples t-test was brought in use to discover the differences within the groups while independent samples t-test was employed to analyze differences between the groups. P-value of 0.05 was the significance level of alpha.

## RESULTS

The demographic characteristics of patients in both groups is shown in Table-I. The mean (±S.D.) age of the patients was 39±5.7 vs. 40±5.3 years in supine vs. prone group respectively. Mean (±S.D.) BMI of the patients was 26±2.5 vs. 25±2.4 kg/m^2^ in supine vs. prone position respectively. There were 22 (77.3%) vs. 21 (70%) male in supine vs. prone group respectively and 8 (26.7%) vs. 9 (30%) female patients in supine vs. prone position respectively. As regard side of symptoms, 18(60%) vs. 19 (63.3%) patients have right side affected in supine and prone group respectively while left side was affected in 12 (40%) vs. 11(36.7%) patients in supine and prone group respectively. ODI and NPRS Score in Patients Having Radiculopathy in Both Groups are shown in Table-II. Mean ODI score was 25.2±6.13 vs. 26.0±6.26 at the start of treatment in supine vs. prone position respectively while it was 19.45±7.12 vs. 11.05±4.40 at end of treatment in supine vs. prone position respectively. Mean NPRS score was 7.73±1.23 vs. 7.67±0.96 at start of treatment in supine vs. prone position respectively while it was 4.63±0.89 vs. 3.13±0.90 at the end of treatment in supine vs. prone position respectively.

**Table-I:** Demographic characteristics of the patients in Both Groups.

Variable	Group A Lumbar traction applied in supine position (Supine)	Group B Lumbar traction applied in prone position (Prone)
***Gender***		
Male	22(73.3%)	21(70%)
Female	8(26.7%)	9(30%)
***Age (years)***		
20 –– 30	4(13.3%)	1(3.3%)
31 –– 40	6(20.0%)	13(43.3%)
41 –– 50	20(66.7%)	16(53.4%)
***BMI (Kg/m^2^)***		
Under weight (BMI < 18.5 kg/m^2^)	0(0%)	0(0%)
Normal(BMI 18.5–24.9 kg/m^2^)	10(33.3%)	16(53.3%)
Overweight(BMI 25.0–29.9 kg/m^2^)	20(66.7%)	12(40%)
Obese(BMI ≥ 30.0 kg/m^2^)	0(0%)	2(6.7%)
***Duration of Disease (in Months)***		
1 –– 12	8(26.7%)	6(20%)
13 –– 24	22(73.3%)	24(80%)
***Side of Symptoms***		
Right	18(60%)	19(63.3%)
Left	12(40%)	11(36.7%)
***Lumbar Root Involved***		
L4 –– L5	4(13.3%)	10(33.3%)
L4 –– S1	8(26.7%)	4(13.3%)
L5 –– S1	18(60%)	16(53.4%)

**Table-II:** ODI and NPRS Score in Patients Having Radiculopathy in Both Groups.

Mean Scores	ODI Mean±S.D.		p-value	NPRS Mean±S.D.		p-value
	
	Supine position	Prone position		Supine position	Prone position	
Baseline score (at week 0)	25.2±6.13	26.0±6.26	0.61	7.73± 1.23	7.67±0.96	0.83
Final score (at week 2)	19.45±7.12	11.05±4.40	0.01	4.63± 0.89	3.13±0.90	0.01

Data in Group-A was seemed to be normal by using Shapiro Wilk Test of normality at 5% level of significance (p=0.24). Data in Group-B was also normally distributed by using Shapiro Wilk Test of normality at 5% level of significance (p=0.32).

## DISCUSSION

The outcome of the study by Filiz MB et al depicted that physical therapy had beneficial effects on modification of pain and disability of patients affected with lumbosacral radiculopathy especially when lumbar traction is applied in prone lying position as compared to supine lying position.^11^ Despite contradictory findings, different physiotherapy maneuvers and exercises with different regimes of physiotherapy modalities have been used to alleviate pain and enhancing functional status of patients with low back pain.^12^,^13^ It is considered that lumbar traction suppresses noxious stimulations by diminishing lordotic curve, expanding intervertebral space and lessening muscle spasm or spinal nerve root pressure.^14^,^15^ Also, in researches by Chung et al^16^ and Ozturk et al^17^ a decrease in the volume of protruded disc has been observed by applying lumbar traction. The results of the study by Krause et al depicted that traction is advantageous for patients with radicular pain concomitant with neurological deficit by causing intervertebral separation.^18^

The supine lying position is generally favored while applying lumbar traction,^18^ Nevertheless lumbar traction can likewise be viably used in the prone lying position.^19^ Patient support and muscle relaxation during traction is viewed as fundamental to accomplish the ideal impacts of the intervention.^20^ Lumbar muscle comfort and quiescence was important to permit spinal distraction, which causes alleviation of “squeezed” spinal nerve roots and decrease of disc herniation.^21^ The electrical activity of paraspinal muscle has been observed in an electromyograghic examination by Kang J et al. In that review, ideal muscle quiescence happened earlier in the prone lying contrasted with the supine lying position. A decrease in the muscle tension was also noteworthy in the prone lying position as compared to supine lying.^22^ As per Tadano S et al, generally mild muscular activation during traction in the prone lying position may permit more noticeable intervertebral separation and accordingly prone lying position might be considered more favorable position than supine lying. Steady with this present researcher contention, better outcomes in regards to pain and disability modification were accomplished by applying traction in the prone lying position as compared to supine lying.^23^

### Limitations of study

There are few significant limitations of this study including scarcity of prior literature comparing supine and prone lying positions for the management of lumbosacral radiculopathy and no follow up later on after completion of study.

## CONCLUSION

Lumbar traction in prone lying position is more effective than lumbar traction in supine lying position for the treatment of chronic lumbosacral radiculopathy.

### Authors’ Contributions:

**RRK:** conceived, designed and did statistical analysis and editing of manuscript.

**RRK, SRA and SRI:** did data collection and manuscript writing.

**SRI and RRK:** did final review and approval of manuscript.
